# Exploring the impact of financial barriers on secondary prevention of heart disease

**DOI:** 10.1186/s12872-017-0495-4

**Published:** 2017-02-14

**Authors:** Kirnvir K. Dhaliwal, Kathryn King-Shier, Braden J. Manns, Brenda R. Hemmelgarn, James A. Stone, David J. T. Campbell

**Affiliations:** 1 Faculty of Nursing, University of Calgary, Calgary, AB Canada; 2 Department of Community Health Sciences, Cumming School of Medicine, University of Calgary, Calgary, AB Canada; 3 O’Brien Institute for Public Health, University of Calgary, Calgary, AB Canada; 4 Libin Cardiovascular Institute of Alberta, University of Calgary, Calgary, AB Canada; 5 Department of Medicine, Cumming School of Medicine, University of Calgary, Health Sciences Centre, G236, 3330 Hospital Drive NW, Calgary, AB T2N 1 N4 Canada; 6 Department of Cardiac Sciences, Cumming School of Medicine, University of Calgary, Calgary, AB Canada

**Keywords:** Coronary artery disease, Secondary prevention, Cardiac rehabilitation, Qualitative research, Financial barriers

## Abstract

**Background:**

Patients with coronary artery disease experience various barriers which impact their ability to optimally manage their condition. Financial barriers may result in cost related non-adherence to medical therapies and recommendations, impacting patient health outcomes. Patient experiences regarding financial barriers remain poorly understood. Therefore, we used qualitative methods to explore the experience of financial barriers to care among patients with heart disease.

**Methods:**

We conducted a qualitative descriptive study of participants in Alberta, Canada with heart disease (*n* = 13) who perceived financial barriers to care. We collected data using semi-structured face-to-face or telephone interviews inquiring about patients experience of financial barriers and the strategies used to cope with such barriers. Multiple analysts performed inductive thematic analysis and findings were bolstered by member checking.

**Results:**

The aspects of care to which participants perceived financial barriers included access to: medications, cardiac rehabilitation and exercise, psychological support, transportation and parking. Some participants demonstrated the ability to successfully self-advocate in order to effectively navigate within the healthcare and social service systems.

**Conclusion:**

Financial barriers impacted patients’ ability to self-manage their cardiovascular disease. Financial barriers contributed to non-adherence to essential medical therapies and health recommendations, which may lead to adverse patient outcomes. Given that it is such a key skill, enhancing patients’ self-advocacy and navigation skills may assist in improving patient health outcomes.

## Background

Coronary artery disease (often referred to simply as heart disease) is a leading cause of morbidity and mortality globally [1]. While some people die following an initial cardiac event, advances in thrombolytic therapy [2] and percutaneous coronary intervention [3], have enabled the vast majority to survive. Given that more people are surviving their first cardiac event, the importance of outpatient management of chronic heart disease is ever growing. A mainstay of heart disease management is the use of proven cardio-protective medications [4, 5]. Another important facet in secondary prevention is engagement in a structured physical activity program, often called cardiac rehabilitation, which has been proven to reduce risk of subsequent events [6].

Many patients with heart disease who should have access to medical therapies do not receive the care that might benefit them. Patients’ access to optimal therapies is hindered by a myriad of barriers, such as physical barriers (e.g., transportation, distance to services) [7], system-level barriers (e.g., wait times [8, 9], access to services [10], and personal barriers (e.g., family responsibilities, personal decision to not seek care) [8–10]. Financial barriers are also commonly experienced among patients with heart disease [11, 12]. Some patients may experience financial barriers directly related to medical therapies (e.g., payments for medications and healthcare team visits) [4, 11, 13], while others experience indirect financial barriers (e.g., employment difficulties, child care costs to allow healthcare provider visits). Patients who experience financial barriers may find themselves unable to adhere to medical therapies [14, 15] or health behaviour recommendations [16] due to direct or indirect costs. Rates of cost-related non-adherence to medications are as high as 10–12% [11, 15] and 23% [14] in Canada and the United States, respectively. Not surprisingly, patient outcomes are impacted by financial barriers and cost-related non-adherence [13], resulting in lower quality of life, poorer overall health status, and increased rate of hospitalizations [11, 12].

As denoted by the Canada Health Act, the Canadian publicly funded health system provides medically necessary hospital and physician services at no direct cost to individual patients. Coverage of outpatient services, including medications, is not universally provided. The degree of coverage and eligibility criteria for these publicly funded benefits varies substantially across provinces [17]. While some heart disease patients may qualify for public benefits, many patients need to purchase private insurance plans to help cover these expenses. Even those who are covered often need to pay substantial premiums, deductibles or copayments to access outpatient medications. While there is important heterogeneity across provinces, our previous studies have found that on average Canadian patients with cardiovascular-related chronic conditions pay approximately $550 per year on drug expenditures, while individuals with several comorbid conditions pay substantially more [11, 18].

Despite the prevalence of financial barriers and their association with important health outcomes in patients with heart disease, their nature and the experience of having financial barriers remain poorly understood. This is particularly concerning as healthcare providers may be unaware of the struggles their patients encounter and may be ill-prepared to help them overcome the root causes of non-adherence to the therapies they prescribe. Being unaware of patients’ individual circumstances and how financial barriers are operationalized in their lives may lead to victim blaming - or blaming non-adherence on personal choices without understanding the constraining context of those ‘choices’ [19]. We thus sought to examine in depth the experience of perceiving financial barriers to care among patients with heart disease by using qualitative methods.

## Methods

### Study design

We undertook a qualitative descriptive analysis of a sub-cohort of participants with coronary artery disease from a broader grounded theory study [20, 21]. Qualitative descriptive studies of this nature have been recognized as important in the field of cardiovascular outcomes research for understanding patient perspectives in complex clinical care settings [22] and when used rigorously can contribute to the advancement of health services delivery models [23].

The purpose of the parent study was to develop a conceptual framework for understanding how financial barriers impact quality of life and clinical outcomes for patients with cardiovascular-related chronic medical conditions (heart disease, diabetes, stroke and hypertension). In this paper we include only the results from the 13 (of 34 total) participants who had self-reported having coronary artery disease. We obtained approval from our institution’s ethics review board and followed recommended procedures and protocols for consent and data collection.

### Sampling and data collection

Full details of sampling and data collection are reported elsewhere [20]. We utilized purposive sampling [24] to obtain a diverse group of participants representing strata that had previously been demonstrated to be associated with perceived financial barriers: age (<65 years/65+), gender, indigenous status, and income [11]. Potential participants were recruited passively via signage in physician offices and pharmacies, as well as actively through research and clinical databases where participants had pre-consented to being contacted for future research studies. To be eligible for inclusion in this study, participants must have identified that they experienced a financial barrier in the past year by answering affirmatively to the following question: *In the past 12 months did you have difficulty paying for services, equipment, medications for your chronic conditions?* The broader study aimed to recruit between 35 and 45 participants which was expected to be sufficient to provide theoretical saturation based on the fact that we were asking questions on a reasonably focused topical area to a group of individuals with similar chronic conditions [24]. Consistent with recommended practices in qualitative research, we collected and analyzed data concurrently. This allowed us to continue sampling and data collection until saturation was achieved in the broader parent study. We pre-determined that saturation would be assumed once three consecutive interviews did not yield any new substantive codes during initial analysis.

We also utilized negative and extreme case sampling to enhance rigor. Extreme cases were individuals who claimed that their financial barrier resulted in a hospitalization in the previous year, while negative cases were individuals who endorsed having financial difficulties but who stated that it didn’t have any meaningful impact on their life.

We collected data using semi-structured interviews that focused on patients’ experiences of financial barriers. Despite some inherent difficulties, our research group has found over the years that telephone interviews using our protocols seem to be effective for gathering this type of data [25]. Participants were therefore offered the choice between on-site face-to-face interview or interview by telephone. Interviews lasted 40–90 min and they were digitally recorded. Recordings of interviews were transcribed verbatim by a professional transcriptionist.

### Data analysis

Transcribed data were imported into NVivo 10 software (QSR International: Doncaster, Australia) for analysis purposes. We used an inductive thematic analysis strategy informed by grounded theory coding techniques [26, 27] All analyses were done in triplicate by three experienced reviewers.

Following data analysis and the development of preliminary findings, member checking (or validating the study findings with a group study participants) [28] was then accomplished by holding a focus group with participants who had previously participated in interviews (*n* = 6). All study participants were invited to participate in the member checking exercise and 6 of 13 participants with heart disease participated. We presented a summary of preliminary findings to participants and received feedback and elaboration on these findings. Data gathered during this focus group was used to consolidate and enhance the findings from the data collection phase of the study.

## Results

The response rate for the broader parent study was 87% (34/39). We interviewed 13 participants with heart disease, 9 men and 4 women (Table 1). The age range was from 47 to 75 years. Most participants identified that they had a prior myocardial infarction (9/13). All participants had comorbid hypertension and/or diabetes. The median number of medications was high: 9 for men and 8 for women. All participants had supplemental health insurance to cover the costs of medications. Most participants were married (9/13), retired or unemployed (10/13), with reported household incomes < $40,000 Canadian dollars (10/13).

**Table 1:** Participant Characteristics

		Men(*n* = 9)	Women(*n* = 4)
Age	Mean (Years)	61.11	60.25
	Range (Years)	47–71	55–75
Heart Disease Type	Angina	3	1
	Myocardial Infarction	6	3
Heart Disease Duration	Mean (Years)	7	4
	Range (Years)	1–20	1–10
Heart Disease Treatment	PCI alone	5	1
	CABG	3	0
	Medical management only	1	3
Comorbid Conditions			
Hypertension	Yes	7	4
	No	2	0
Diabetes	Yes	6	3
	No	3	1
Stroke	Yes	0	2
	No	9	2
Seen a medical specialist in the previous year	Yes	8	3
	No	1	1
Number of medications	Median	9	8
	Range	6–13	5–12
Supplemental Health Insurance	Employer-Sponsored	2	1
	Publicly Funded	4	2
	Non-Group*	3	1
Marital Status	Married/Common-law	7	2
	Separated/Divorced/Single	1	2
	Widow/Widower	1	0
Education	High School or less	3	3
	Some Post-Secondary	5	1
	Bachelor’s Degree or Higher	1	0
Employment status	Employed	2	1
	Retired	5	1
	Unemployed	2	2
Income category	<$20,000	2	2
	$20 – 40,000	4	2
	>$40,000	3	0

*Non-Group coverage is that purchased privately by those not otherwise insured

Despite having a universal single payer healthcare system in Canada, we learned that heart disease patients experience significant financial barriers to various aspects of care. These may hinder patients from accessing crucial healthcare services, and potentially contribute to worse health outcomes. The most prominently described aspects of care to which participants perceived financial barriers included access to: medications, cardiac rehabilitation and exercise, psychological support, and transportation/parking costs.

### Medication access

Medical therapies are critical to preventing future cardiovascular events. Nearly all participants raised concerns regarding the affordability of their medications. Unfortunately, many participants reported that paying for their required cardioprotective medications consumed much of their budgets: *“You kinda scrimp and save and pull all your resources together and then the cost of your medication just about gobbles that up.”* (Male, P2). Similarly, another participant stated about her medications: *“well look at how much money I would save”* (Female, P8).

Several participants stated that their financial barriers resulted in cost-related medication non-adherence: *“The more you burden people with the medical costs the worse off they are. They can’t concentrate on getting better. Let’s face it, I’m sure it’s well known that people don’t take their pills. They can’t afford to.”* (Male, P3) In the worst cases, some participants identified that their cost-related non-adherence led to adverse clinical outcomes: *“I was off them for 3 months and I really didn’t feel any difference. And then when you end up in hospital and realize that maybe I should’ve been taking them, kind of an eye opener but it’s an everyday struggle.”* (Male, P5)

Participants discussed various strategies to overcome their financial barriers with respect to medications, including: prioritizing some medications over other needs; obtaining additional insurance; and the use of government programs. Some participants ‘separated’ their medications into essential and non-essential categories. For example one participant said: *“Well my medication, my diabetes, the Metformin, I’ll continue buying that but the rest of it will go by the wayside.”* (Male, P2) Unfortunately, some participants had to prioritize their medications over other critical preventive health needs, such as healthy food: *“And that’s something that I have to do… if it’s medication or food, well it’s gonna have to be the medication in order to live and to get through this.”* (Male, P5)

Despite the fact that all respondents had supplemental health insurance, often sponsored by the government or employers, only a small number of participants reported being adequately insured. Many of those who had insurance reported substantial out-of-pocket costs in the form of copayments: *“Part of my prescriptions are paid by my company but still it’s an expense… [For] a lotta people it’d be like $135, wow, that’s not very much but for me it’s still a lotta money.”* (Female, P4) Beyond copayments, for those with unsponsored (non-group) insurance, premiums may be an issue as well. Several participants identified that their premiums cost more than the benefits they provide. For example, a participant reported paying $2000 in premiums for $1800 worth of drug expenses.

Some participants identified that government income support programs (which generally also include full coverage for medications) helped them overcome financial barriers: *“If it wasn’t for me getting on [government program] and getting help with medication… it was costing me over $500 a month.”* (Male, P2)

### Cardiac rehabilitation and exercise

Physical activity, especially in the context of a structured cardiac rehabilitation (CR) program is important to minimize the risk of subsequent cardiovascular events. In this study, participants universally described being referred to a CR program following hospital discharge, and being encouraged by healthcare providers to engage in physical activity. Unfortunately, for many, participation in exercise activities was hindered by the cost: *“The downside of physical activity (is) I can’t afford to even do that.”* (Female, P7)

In Alberta, there can be considerable direct fees for CR patients, however these can be waived if the patient states that they are unable to pay. Unfortunately, several participants were unaware of the fee waiver program and expressed concerns regarding the fees: *“I was shocked, like absolutely jaw dropping shock when I heard that it was gonna be like the huge amount, that it was gonna be”* (Female, P4) and *“It was never said that there’s financial assistance if you need it.”* (Male, P3) Another participant was advised of the program, but stated: *“The fee…they said it could be like a sliding scale. But I assumed that it couldn’t slide that far down for me.”* (Female, P4) One participant recounted the shame experienced from the inability to pay: *“I’ve never needed that kind of help forever and then all of a sudden you’re saying I have to go to this cardiac rehab, it’s a good program but I can’t afford to pay for it. So yeah, that kinda, it hits home…a little embarrassed I guess.”* (Male, P3)

Other than the direct cost of the program, several participants perceived financial barriers incurred due to the required time away from work to participate in CR:

*“When you do the simple math it’s like, ‘oh well we want you here for 12 weeks, 2 times a week, 3 h a day’… you lose an hour in the morning, you exercise an hour, an hour to get back to work. Three hours a day, 6 h a week, 12 weeks, 72 h. $25 an hour, plus the $500 that they don’t cover in the exercise program. That’s pretty close to $3,000 to me. I’m sorry but I’m not doing their exercise program.”* (Male, P5)

While the fee waiver program did not work for all respondents, many were able to enrol in this program: *“I told them I didn’t have any money…I said there’s absolutely no question that I can’t afford any of this and they said ‘okay then’. They said ‘don’t worry about it, we’ll waive it’”.* (Male, P9)

Several participants expressed that they were very happy with the CR program they attended, but that they experienced financial barriers to ongoing physical activity after having completed their 12-week CR program: *“I gotta buy a membership. Well I can’t afford memberships.”* (Male, P10) Another respondent stated: *“We have got fees, you know, you have got charges to pay…about $60 a month or something.”* (Male, P11) and: *“[My doctor] wants me to do aquasizing. Well I can’t afford that… it’s not in the cards to even do the pool. I would love to but nope”*. (Female P7)

### Psychological support

It is well known that patients with heart disease are at risk of significant mental and emotional health difficulties. Many participants expressed that this was exacerbated by their inability to work due to their heart disease and that the subsequent need to rely on others was taxing on their self-esteem:

*“When you work you’re whole, but when you get to the point of having to depend on other people for your income… It’s like you don’t become a whole person anymore. You become pieces. And if you don’t have that piece to help you through that life you can’t be whole. It’s like you’re lost.”* (Female, P7)

Social isolation resulting from financial barriers also contributed to participants’ feeling depressed. Most participants sacrificed social activities with the goal of saving money to spend on their healthcare requirements (e.g., medication costs). For example, several participants reported avoiding visiting the movie theatres, eating at restaurants, and other recreational activities. A participant stated: *“I can’t remember the last time I went out. I’m in my apartment 7 days a week. So you do miss out on interactions with other people…you have no social life because you can’t afford it.”* (Female, P4)

Some participants identified that their financial barriers were not simply a cause of psychological distress, but that these also hindered them from being able to access necessary psychological supports. A participant stated: *“I really could’ve used more emotional support from the system in terms of you know, ‘you’ve had a heart attack, how do you feel? How do you feel about life? To be able to discuss these issues of being confronted with one’s own mortality.”* (Male, P9) One participant explicitly stated: *“So if I would’ve had some money to be able to afford (it) I probably would’ve went and got some professional help.”* (Male, P3)

### Transportation and parking

The incidental costs of seeking healthcare, such as transportation and parking were a barrier that hindered some participants’ access to medical care. Transportation related issues included: not being able to drive independently, having prohibitively long distances to healthcare facilities, costs of gasoline, and the cost of parking at hospitals and doctors’ offices.

Numerous participants described incurring indirect costs due to driving long distances to healthcare facilities, such as one participant who lived far from the regional CR program*: “You gotta run all the way to [the city] and that’s ¾ of an hour at very best…one way, so I mean you lose your day.”* (Male, P6) Others described that the cost of gasoline was prohibitive to them following up as directed: *“I had to pay my gas to drive into town and everything. You gotta drive an hour, over an hour to the [rehab centre] to go to cardiac rehab twice a week? That is a huge financial burden on people”* (Male, P3)

Finally, a commonly cited transportation-related financial barrier was related to the high cost of parking at healthcare facilities. Many participants stated that they had multiple specialist appointments to attend, and these each required them to spend significantly for transportation and parking:

*It’s just a strain. This week we had Dr. [cardiologist] this morning at 8:15 and I had an 11:15 at the [Hospital] and was there ‘till quarter after 2. Tomorrow I have the health nurse coming in and then I have to drive all the way up to the [other hospital] to see the vascular surgeon.* (Female, P13)

Unfortunately, participants reported avoiding healthcare services (e.g., CR) because of transportation related issues: *“I started [CR] and it was going to be way too much travel for me.”* (Female, P4)

### Navigation and advocacy

Clearly, when one is faced with financial constraints it is important to be able to access resources that may be available. Many participants acknowledged that one main problem was difficulty navigating the healthcare system and/or advocating for themselves to obtain the information or assistance they required. The perceived causes of these difficulties stemmed from a lack of understanding of the complexities of the healthcare system or processes and an inability to communicate effectively with healthcare providers.

For example, a participant who recently moved from another province reported encountering difficulties identifying resources: *“It’s been hard. I still don’t know all the resources… everything is so split up here.”* (Female, P7)

Being able to effectively communicate with healthcare providers is a key navigation skill to overcome and deal with an individual’s financial barriers. One participant felt unwell for some time following his hospital discharge, he stated: *“So after about a year I got a bit angry with the cardiologist and said look, there’s a problem here, I want you to check what it is and fix it. And he kinda looked at me like who do you think you’re talking to.”* (Male, P1) This participant’s ability to advocate for himself led the cardiologist to order further testing and adjust his medications accordingly.

By contrast, another participant expressed concerns that he had multiple appointments with various providers, yet believed he was lacking appropriate attention from any of them. He said:

*“It’s frustrating not to be followed but at the same time you gotta go to a doctor every week. I really struggle and fight with that fact, it’s like, nobody’s bothering to keep a check on any of this.”* (Male, P5)

Other participants praised the healthcare providers that were able to help them navigate through the healthcare and community support system: *“I mean these guys that I’m dealing with now with my heart problems… I’ve got nothing bad to say about these guys. They bent over backwards to get me back on [social programmes]. The nurses are real good to deal with.”* (Male, P12)

Clearly, there was a ‘skill set’ that some participants demonstrated in navigating the healthcare system and advocating for their needs which enabled them to overcome financial barriers which was lacking for numerous others.

## Discussion

Given the growing numbers of patients and the known societal costs associated with managing chronic heart disease [11, 12], we undertook this qualitative descriptive study to explore patients’ experiences with financial barriers. Patients in this study identified that limited financial resources reduced their ability to access medications, exercise/CR and psychosocial support (all of which are known, when not available, to increase the risk of future coronary events) [29].

Financial barriers are commonly experienced among patients with heart disease [11, 12]. A component of heart disease management is the use of proven cardio-protective medications; [4] greater than 90% of Canadian patients with multiple chronic diseases take prescription medications [5]. Our study participants raised concerns regarding the affordability of their medications, with several admitting to engaging in cost-related non-adherence. Our findings are consistent with previous studies that report cost-related medication non-adherence is relatively common in this population [11, 15]. Some participants identified their cost-related medication non-adherence led to adverse clinical outcomes. This is in keeping with prior studies that reported patients who experience financial barriers encounter severe clinical consequences more frequently (e.g., poorer health status, and higher rates of all-cause re-hospitalization, cardiac re-hospitalization, and mortality) [12, 30]. This is unsurprising given that medication nonadherence has been independently associated with these adverse outcomes and higher costs of care [31, 32].

All study participants reported having health insurance, yet they still encountered substantial out-of-pocket costs (e.g., copayments and premiums). Our finding is consistent with prior studies that insurance coverage may not eradicate financial barriers because patients may remain underinsured and faced with substantial copayments [33]. Not surprisingly, patients who are underinsured are more likely to engage in cost-related medication nonadherence [15]. This is an important finding as these barriers are potentially modifiable given that insurance coverage is amenable to being addressed by changes in public policy [34].

Cardiac rehabilitation has been proven to reduce risk of subsequent cardiac events [6, 35]. Numerous participants in our study reported cost as a barrier to accessing CR and post-CR exercise. Qualitative metasyntheses have identified “accessibility” and “financial and work constraints” as barriers to CR attendance [36, 37]. Similarly, several studies have identified low socioeconomic status [38, 39] and cost [40] as barriers to CR participation.

Participants reported that mental and emotional health difficulties were exacerbated by their inability to work due to their heart disease. Furthermore, we identified that social isolation resulting from financial barriers contributed to participants’ feeling depressed. Several authors have reported that psychosocial risk factors, such as lack of social support; social isolation; and depression, negatively contribute to the prognosis and recovery from cardiovascular disease [40, 41].

The incidental costs of seeking healthcare, such as transportation and parking were a barrier that hindered some participants’ access to medical care. For example, participants reported avoiding healthcare services (e.g., CR) because of transportation related issues. Transportation related issues included: not being able to drive independently, having prohibitively long distances to healthcare facilities, costs of gasoline, and the cost of parking at hospitals and doctors’ offices. This finding has been commented on before by other authors who have identified that lack of transportation [35, 38] and long distances to services [37] may be barriers to CR participation.

Many participants experienced difficulty navigating the healthcare system and/or advocating for themselves to obtain the information or assistance they required. The perceived causes of these difficulties stemmed from a lack of understanding of the complexities of the healthcare system and an inability to effectively communicate with healthcare providers. In contrast, some participants were able to effectively advocate for themselves and communicate with healthcare providers. Being able to effectively communicate with healthcare providers is a key navigation skill to overcome and deal with financial barriers. Clearly, some participants demonstrated a ‘skill set,’ specifically effective navigation and self-advocacy skills, which enabled them to overcome financial barriers. Likewise in one study, participants who actively sought health information were more likely to be involved in their healthcare [42]. Patients recognize the importance of self-advocacy and have described self-advocating behaviour [43], which may impact their ability to navigate within the healthcare system. For example, when a patient is able to share concerns regarding medication costs with a healthcare provider (e.g., pharmacist, physician, nurse, social worker), information regarding support programs can be offered by the provider and accessed by the patient [44]. The barrier to accessing these goods and services is therefore reduced. Healthcare providers may need to assist patients in developing self-advocacy skills [43, 45, 46], in an effort to promote successful navigation.

There were both strengths and limitations to this work. The people who participated in this study were keenly interested in sharing their experiences about having financial barriers to care. A major strength of this work was that interviewers were well trained in qualitative interviewing techniques. Thus, we believe that the participants were candid and authentic with their responses to our questions. We also used rigorous qualitative methods including member checking and multiple analysts to improve the trustworthiness of our findings. There were some limitations to this work as well. Because the interviews were undertaken at only one point in time, we were unable to determine how the participants’ experiences may change over time. We acknowledge that there may also be concerns about the transferability of the study findings, given the small sample size from one geographic location. However, the number of participants in this study was in keeping with similar qualitative studies. Furthermore, it is important to note that low socioeconomic status is strongly associated with coronary artery disease, therefore [47] many heart disease patients are at high risk for having financial barriers. This significantly increases the likelihood that our findings are representative of and transferable to a large proportion of patients with coronary disease across internationally. While conducting this analysis, it was our impression that we had achieved some degree of saturation as similar themes appeared in each transcript. However, while sampling for the broader parent study continued until saturation was reached, because this study is a secondary analysis we cannot say with certainty that the heart disease cohort was fully saturated.

## Conclusion

Patients with coronary artery disease face numerous barriers that prohibit them from accessing the care they require for optimal secondary prevention. In this qualitative study, we explored the facets of care to which financial barriers are most likely to arise: medications, physical activity (including cardiac rehabilitation), psychosocial supports and transportation.

Self-advocacy and navigation skills were key factors that some patients may use to overcome their financial barriers. This is an important finding, as this is a potentially modifiable factor which healthcare systems and individual healthcare providers can foster in patients to mitigate the impact of financial barriers on coronary disease patients.

## Abbreviations

CR: Cardiac Rehabilitation
